# A New Non-invasive Technique for Measuring 3D-Oxygen Gradients in Wells During Mammalian Cell Culture

**DOI:** 10.3389/fbioe.2020.00595

**Published:** 2020-06-17

**Authors:** Carlos J. Peniche Silva, Gregor Liebsch, Robert J. Meier, Martin S. Gutbrod, Elizabeth R. Balmayor, Martijn van Griensven

**Affiliations:** ^1^cBITE, MERLN Institute for Technology-Inspired Regenerative Medicine, Maastricht University, Maastricht, Netherlands; ^2^PreSens Precision Sensing GmbH, Regensburg, Germany; ^3^IBE, MERLN Institute for Technology-Inspired Regenerative Medicine, Maastricht University, Maastricht, Netherlands

**Keywords:** oxygen, gradient, respiration rate, non-invasive, ramp, optical-based, sensor foil

## Abstract

Oxygen tension plays an important role in overall cell function and fate, regulating gene expression, and cell differentiation. Although there is extensive literature available that supports the previous statement, little information is to be found about accurate O_2_ measurements during culture. In fact, O_2_ concentration at the cell layer during culture is commonly assumed to be equal to that of the incubator atmosphere. This assumption does not consider oxygen diffusion properties, cell type, cell density, media composition, time in culture nor height of the cell culture medium column. In this study, we developed a non-invasive, optical sensor foil-based technique suitable for measuring the 3D oxygen gradient that is formed during cell culture as a result of normal cell respiration. For this propose, we created a 3D printed ramp to which surface an oxygen optode sensor foil was attached. The ramps were positioned inside the culture wells of 24 well plate prior cell seeding. This set up in conjunction with the VisiSens TD camera system allows to investigate the oxygen gradient formation during culture. Cultivation was performed with three different initial cell densities of the cell line A549 that were seeded on the plate containing the ramps with the oxygen sensors. The O_2_ gradient obtained after 96 h of culture showed significantly lower O_2_ concentrations closer to the bottom of the well in high cell density cultures compared to that of lower cell density cultures. Furthermore, it was very interesting to observe that even with low cell density culture, oxygen concentration near the cell layer was lower than that of the incubator atmosphere. The obtained oxygen gradient after 96 h was used to calculate the oxygen consumption rate (OCR) of the A549 cells, and the obtained value of ~100 fmol/h/cell matches the OCR value already reported in the literature for this cell line. Moreover, we found our set up to be unique in its ability to measure oxygen gradient formation in several wells of a cell culture plate simultaneously and in a non-invasive manner.

## Introduction

Oxygen tension plays an important role in regulating gene expression, cell differentiation and overall cell function and fate (Wenger, 2000; D'Ippolito et al., 2006; Hirao et al., 2007; Ivanovic, 2009; Vander Heiden et al., 2009; Rankin et al., 2011; Wagner et al., 2011; Nicolaije et al., 2012; Al-Ani et al., 2018). During bone formation, hypoxic gradients, that is an oxygen concentration range from 5 to <1%, are established as the tissue grows. These gradients promote the formation of new blood vessels that deliver oxygen and nutrients to the growing tissue (Rankin et al., 2011). During cell culture *in vitro*, oxygen tension below 2–3% has been related to the inhibition of the osteogenic differentiation of human bone marrow-isolated multilineage inducible cells, while similar oxygenation conditions stimulated matrix mineralization and accelerated the transformation of osteoblasts to osteocytes in cultures of MC3T3 (an osteoblast precursor cell line) (D'Ippolito et al., 2006; Hirao et al., 2007). In immune cells, low oxygen tensions have been proven to affect the immune response by down-regulating T-cells function. For example, *in vitro* studies have shown that low O_2_ concentration causes prolonged impairment of cytokine expression. Oxygen tension also affects the balance between T helper 1 cells and T helper 2 cells. For instance, low oxygen tension causes a shift toward T helper 2 responses and inhibits the T helper 1 responses (Sitkovsky and Lukashev, 2005). Furthermore, decreased oxygen tension (≤ 5% oxygen concentration) also inhibits the capacity of mesenchymal stem cells to differentiate (Al-Ani et al., 2018) while higher oxygen tension values have been reported to promote differentiation (Ivanovic, 2009).

The previously mentioned facts illustrate the relevance of oxygen tension on how the cells react to their environment. In *in vivo* conditions, oxygen levels are finely tuned with respect to tissue and cell type by means of highly complex mechanisms that, until now, can't be replicated during *in vitro* cell/tissue culture. The oxygen concentration to which tissue is exposed in *in vivo* conditions are much lower than that of the atmosphere, even in those tissues in direct contact with air (Al-Ani et al., 2018). In contrast, culture of cells in incubators having ambient atmosphere, is often referred to as “normoxia,” while cultures in incubators with lower levels of oxygenation are referred to as “hypoxia” (Saltzman et al., 2003; Wild et al., 2005; Wenger et al., 2015). In particular, “normoxic” incubators are erroneously assumed to deliver 20.9% of oxygenation to the cells in culture without considering other parameters, such as medium diffusion properties, height of the cell culture medium column, cell density and oxygen consumption rate (Wenger et al., 2015; Al-Ani et al., 2018). Another aspect to consider is that the oxygen concentration in the gas phase of a “normoxic” incubator at sea level is actually 18.6% (Wenger et al., 2015). The reason for this fact is that the gas mixture inside an incubator differs from that of the atmosphere in the content of N_2_, O_2_, H_2_O, and CO_2_ due to the extra content of CO_2_ (38 mmHg for a 5% v/v concentration) and water vapor (47 mmHg) found inside an incubator, which is necessary for the maintenances of stable pH and the appropriate humidified conditions during cultivation, respectively. According to Dalton's law, the partial pressure of the gases inside a normobaric incubator will sum up to equal the atmospheric pressure outside the incubator, which at sea level is 760 mmHg. This means that the actual pO_2_ inside an incubator at sea level, when considering the contribution of the partial pressure of the extra CO_2_ and water vapor, is 141 mmHg, equivalent to 18.6% of the total atmosphere of the incubator.

Due to the essential role of oxygen in almost every biological process, inaccurate oxygen concentration measurements during cell culture could greatly affect the reproducibility of the experimental results. This also applies when the importance of monitoring the oxygen concentration during cell culture is underestimated (Karp, 2018).

Over the years, a broad spectrum of techniques has been explored for measuring oxygenation during cell culture. For instance, solid state electrodes, such as the Clark-type electrode, have been used for oxygen sensing during cultivation. These electrodes work by reducing oxygen electrochemically and recording the changes in current (Renger and Hanssum, 2009; Strovas et al., 2010). The main drawbacks of this system include oxygen consumption by the electrode itself. This effect is usually negligible in large volumes but it can limit Clark type electrodes applications in low volumes. This is why, in recent years many electrochemical oxygen sensors have been replaced by optical counterparts (Randers-Eichhorn et al., 1996; Strovas et al., 2010; Demuth et al., 2016). Alternatively, recent methods involving scanning electrochemical microscopy (SECM) and nanobead sensors attached to the outer membrane of cells have been employed (Saito et al., 2006; Kuang and Walt, 2007). Nonetheless, these techniques are only able to measure oxygen concentration near the cell membrane or near the oxygen sensor.

Techniques based in optical methods for measuring oxygen concentration quickly gained popularity because of their accessibility and their ability to indicate real time oxygen concentration in a non-invasive fashion (Randers-Eichhorn et al., 1996; Kellner et al., 2002; Strovas et al., 2010; Wang and Wolfbeis, 2014). The vast majority of optical sensors for oxygen are based in luminescent probes consisting of a transparent support and a sensing layer. This layer usually contains a quenchable probe and an unquenchable reference dye (Wang and Wolfbeis, 2014). Some techniques combine fluorescent sensor foils with 2D read-out technology to measure the pericellular oxygen concentration (Tschiersch et al., 2012; Wolff et al., 2019). With this system it is possible to gather real time information about the oxygen levels near the oxygen sensor. However, the amount of information that can be obtained using this system has been until now, limited to the oxygen concentration over a cross section of the cell culture and does not provide any data about the oxygen gradient inside the cell culture medium.

On the other hand, the use of an integrated needle-type micro sensor and recording system has allowed the measurement of the oxygen concentrations not only near the surface of the cells in culture but also the oxygen distribution inside the cell culture medium (Pettersen et al., 2005). With this technique it is possible to study the oxygen gradient formation that occurs during cell culture and its relation with cell density (Malda et al., 2004; Pettersen et al., 2005; Wenger et al., 2015). However, practical limitations for this technique restrict its use to a single culture well or T-Flask at a time. This greatly affects the applications of needle-type micro sensors in terms of simultaneous oxygen measurements due to the fact that any micro sensor multiplexing results in a quick rise of cable management complexity. In addition, the necessary set up for this technique involves the needle-type sensor moving inside the cell culture medium and near the cell surface. This means that the well plate or flask needs to be left open during the measurement. This endangers the sterility of the culture, especially when several sequential measurements are needed (Randers-Eichhorn et al., 1996).

**Figure 1:**
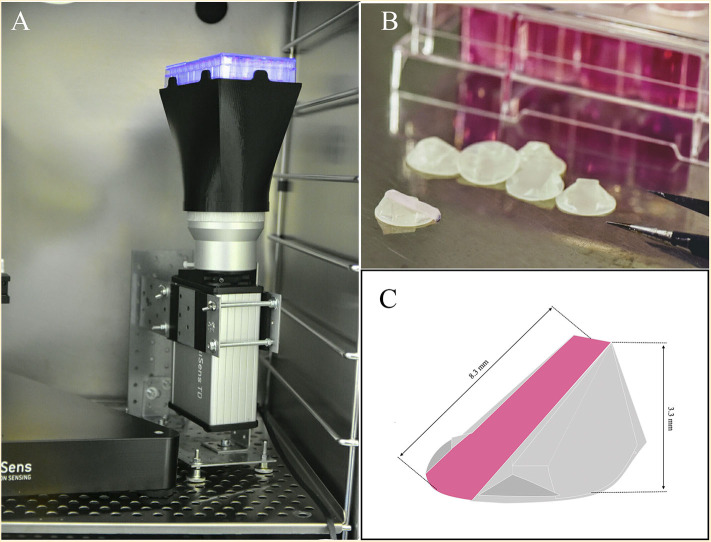
**(A)** Set-up of the VisiSens TD camera system used during the measurements. A cell culture plate is mounted on the plate support/base on the camera system. A light source located around the camera lens illuminates the cell culture plate from below to excite the oxygen sensor foils inside the wells. The VisiSens TD camera collects the ratiometric signal from the dyes on the surface of the sensors as they interact with the dissolved oxygen inside the cell culture media. **(B)** Image of the ramps with the sensors attached on their surface. **(C)** Schematic representation of the 3 D printed ramp and geometric details. The SF-RPsSu4 sensor was attached to the rectangular surface of the ramp.

Therefore, we aimed to develop a novel intrinsic system to measure oxygen gradients over time during cell culture. We have developed a simple and cost-effective technique that allows the non-invasive, real time study of the 3D oxygen gradient formation during cell culture. We based our technique on the use of the VisiSens TD camera system and the planar oxygen sensor foil SF-RPsSu4 (Maisch et al., 2019; Wolff et al., 2019). We created a 3D printable ramp to which the SF-RPsSu4 sensor can be attached on its diagonal surface (Figure 1) and can be incorporated inside cell culture wells. This configuration allows the sensor to measure the oxygen concentration at different heights from the bottom of the well to a maximum of 3.3 mm over the bottom without any moving parts involved in the set up. We also compared the oxygen profiles obtained with our developed technique to that obtained with the optical fiber-based needle type sensor PM-PSt7 mounted on a motorized and computer-aided micromanipulator. Our results show that with our set-up, oxygen gradient formation can be measured simultaneously in several wells of a cell culture plate in a non-invasive manner.

## Materials and Methods

### Cells and Culture Conditions

Immortalized human lung epithelial cell line A549 (ATCC®-CCL-185™, ATCC, Manassas, VI, USA) were cultured in high glucose Dulbecco's Modified Eagle's Medium (D-MEM, Sigma-Aldrich, St. Louis, MO, USA) supplemented with fetal bovine serum (FBS, 10%) and a mixture of penicillin 100 U/ml and streptomycin 100 μm/ml (1%, Sigma-Aldrich, St. Louis, MO, USA).

Cultivation and oxygen concentration measurements were conducted under standard culture conditions in a humidified atmosphere containing 5% CO_2_ and 18.6% O_2_ at 37°C. No media exchange was conducted during the measurements.

### Non-invasive Oxygen Concentration Measurement at Cell Monolayer

The VisiSens TD camera system (PreSens, Regensburg, Germany) was employed to monitor the oxygen concentration at the cell layer. The setup of the VisiSens TD camera system is presented in Figure 1A. Images were taken every hour for a period of 96 h. The VisiSens TD system works in conjunction with the commercially available, biocompatible, planar and self-adhesive oxygen-sensitive foil SF-RPsSu4 (PreSens, Regensburg, Germany).

This sensor exploits a fluorescence optical sensing scheme It contains an oxygen sensitive dye which reversibly interacts with the analyte via dynamic quenching (Wang and Wolfbeis, 2014; Wolff et al., 2019) and changes its phosphorescence intensity. Furthermore, the sensor foil includes an analyte insensitive reference dye. Both dyes are simultaneously excited via an LED lamp mounted on the VisiSens TD camera detector. The camera detector records both emitted luminescence signals (red emission from the indicator and green emission from the reference dye) spectrally separated in its RGB color channels. Each recorded color pixel contains the local indicator and reference signal and thereby allows for ratiometric 2D RGB imaging (Tschiersch et al., 2012). The obtained set of images can be loaded into the software ScientifiCal (PreSens, Regensburg, Germany) and the color recorded to each pixel is converted to air saturation (%) by a two point calibration step (0% and 100% air saturation).

The calibration curve for this system follows an adapted Stern-Volmer (Klimant et al., 1995).

$$\begin{array}{l} \frac{Io}{I}=\frac{Ro}{R}=\frac{\tau o}{\tau }={\left(\left(\frac{A}{1+ksv^{*}pO2}\right)+\left(1-A\right)\right)}^{-1} \end{array}$$

The written equation describes decay time (τ), intensity (I), intensity-ratio (R) in analogy, the index 0 describes the time, intensity or ratio at 0% O_2_ (calibration point at 0% air saturation)

The sensor constants to mathematically describe the exact behavior of the oxygen dependent response curve are factory calibrated for the material. The non-linearity is a fixed material property of the sensing material (represented by parameter A). The value A for the used SF-RPSu4 oxygen sensor foil is give as 0.82. The two-point user calibration for 0 and 100% air saturation alongside with the factory calibrated sensor constants allow for precise and accurate determination of oxygen with these sensors.

For the measurement, the SF-RPSu4 sensors were cut manually to a diameter of 10 mm and glued to the bottom of the wells of a 48 well plate (Eppendorf AG, Hamburg, Germany). The SF-RPSu4 sensors are commercially available with biocompatible self–adhesive backside. The glue is a pressure sensitive adhesive that requires no curing time nor waiting time before the sensors are applied. One-time incubation for 30 min with 70% ethanol was conducted for disinfection of the sensors. Afterwards, ethanol was aspirated and the sensor foils were allowed to rest for 30 min to ensure the evaporation of the remaining ethanol. Subsequently, sensors were washed twice with PBS (Sigma-Aldrich, St. Louis, MO, USA). Conditioning of the sensor foils was performed by 1-h incubation with cell culture medium at 37°C to enhance cell adhesion as suggested by the manufacturer. A total of 7 × 10^4^ A549 cells/cm^2^ were seeded on the attached sensors in triplicate.

Triplicates of wells containing only the sensor foils and cell culture medium were included as negative controls. Two single wells equipped with SF-RPSu4 sensors were used for a two-point calibration (0 and 100% O_2_). For the 0% O_2_ calibration, a solution of 5% sodium sulfite (Na_2_SO_3_) was prepared. The oxygen dissolved in the cell culture medium is consumed by reacting with the sodium sulfite to form sodium sulfate (Na_2_SO_4_). Oxygen saturated cell culture medium was added in the second well to obtain the 100% air saturation calibration point (equivalent to 20.95% O_2_).

The cell culture plate was mounted on the VisiSens immediately after cell seeding and images were taken every hour during 96 h. All the images taken from the same experiment were loaded to the software VisiSens ScientifiCal (PreSens, Regensburg, Germany) as one single series (stack) of images. Noise reduction was applied with the standard value of 20 simultaneously to all the images from the series and all times images were processed in the same way. The noise reduction function is a color threshold that discards all pixels from the measurement that show too low intensity values. These noise filtered pixels represent the areas where no sensor is present in the recorded field of view, so that no fake pixels were taken into account for calculation. Calibration was performed by assigning the 0% O_2_ and 100% air saturation references values to the wells containing the respective calibration solutions. A Z-profile tool was employed to manually select the region of interest (ROI) directly on the wells containing the cells seeded on the SF-RPsSu4 sensor foils. This tool allows the determination of the average value of air saturation from each recorded pixel within the selected ROI per image. Values expressed as air saturation percentages were converted to oxygen saturation considering that 100% air saturation equals 20.95% oxygen saturation at 37°C.

### Non-invasive Oxygen 3D Gradient Measurement

The resin YOE-Microflu 365 (Miicraft, Burms, Germany) was utilized for producing the 3D printed ramps with the use of the MiiCraft DLP 3D printer (Ray Optics, Taiwan). The ramps were designed to be 8.3 mm long reaching a maximum height of 3.3 mm above the bottom of the well (Figures 1B,C). With a surface area on the base of the ramp of 54 mm^2^, the ramp occupies 27% of the surface area available for cell growth in a well from a 24 well plate.

The VisiSens TD camera system was employed to investigate the 3D oxygen gradient formation during 96 h of culture with a time interval of 1 h between two measurements. The SF-RPsSu4 sensor foils were cut by hand and attached to the rectangular-shaped surface of the ramp. The ramps were randomly positioned at the bottom of each well prior cell seeding. Sterilization was conducted as described above for the monolayer experiments.

A total of 5.0 × 10^4^; 1.0 × 10^5^ and 1.5 × 10^5^ A549 cells were seeded per well in a 24 well plate (Eppendorf AG, Hamburg, Germany) in triplicate. This is, 0.35 × 10^5^, 0.68 × 10^5^, and 1.02 × 10^5^ cells/cm^2^, respectively considering the surface area occupied by the ramp inside the culture well. The final volume of cell culture medium in each well was 1 ml, leaving the ramps totally submerged.

The cell culture plate containing the ramps with the attached sensor foils, calibration solutions, and cells was mounted on the VisiSens immediately after seeding and images were taken every hour during 96 h.

Another three 24 well plates were prepared as described before and used for obtaining the cell count for three time points of interest; 48, 72, and 96 h. Manual cell count was performed by using a Neubauer chamber using the exclusion dye Trypan blue.

**Figure 2:**
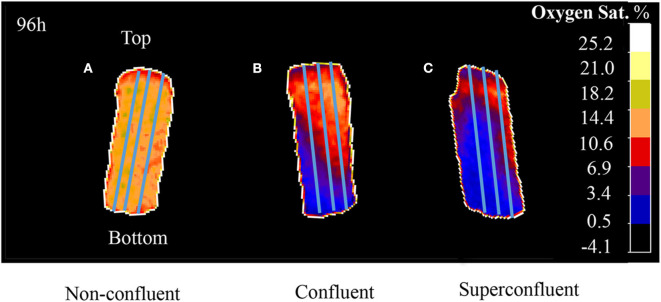
A profiling tool was use to draw triplicates of a line across the entire length of the sensor (from the bottom to the top of the ramp). VisiSens ScientifiCal output for three different degrees of culture confluence: **(A)** subconfluent, **(B)** confluent, and **(C)** superconfluent after 96 h of culture. Initial cell densities used were 3.5 × 10^4^ cells/cm^2^ for subconfluent, 6.8 × 10^4^ cells/cm^2^ for confluent, and 1.02 × 10^5^ cells/cm^2^ for superconfluent cultures.

For the 3D oxygen gradient measurement, the images corresponding to the time points 48, 72, and 96 h were selected. A lineal profiling tool was employed to measure the oxygen concentration values across the length of the sensor foil per time point. For this, triplicates of a profiling line were drawn from the bottom to the top of each ramp (Figure 2) for each one of the selected time points.

### Determination of the Oxygen Consumption Rate

Oxygen consumption rate (OCR) was determined after 96 h of culture for those wells where a theoretical linear oxygen gradient was observed to be formed. That is, where the measured oxygen gradient fits a straight line with *R*^2^ > 0.9. According to Fick's first law of diffusion, a solute will move from high concentration zones to low concentration zones with a magnitude proportional to its concentration gradient. The mathematical equivalent to Fick's first law is the equation $J=-D\frac{\Delta C}{\Delta x}$ where “J” represents the flux of a solute, “D” the diffusion coefficient of the solute in the mixture and ($\frac{\Delta C}{\Delta x}$) the spatial concentration gradient of the solute determined experimentally.

During cell culture, normal cell respiration at the bottom of the wells creates a low oxygen concentration zone compared to the surface of the well. This results in a concentration gradient that, assuming steady state conditions, will be the only driving force for the flux of oxygen from the top to the bottom of the well (Pettersen et al., 2005).

In our experiments, the absence of movements or turbulences in the cell culture media and the constant atmospheric pressure inside the incubator implies a steady state-like condition, where the diffusion of oxygen to the cell layer is driven only by the oxygen consumption rate of the cells in culture Therefore, it is safe to assume that the flux of oxygen will be equal to the respiration rate of the cells in culture (Pettersen et al., 2005). The diffusion coefficient of oxygen (D) in cell culture medium (9% salinity and 37°C) is ~3.37 × 10^−5^ cm^2^/s (Pettersen et al., 2005). Cell count after 96 h of culture was determined from one of the plates prepared for this propose as described before.

### Oxygen Profiling With Fiber-Based Needle Type Microsensor

**Figure 3:**
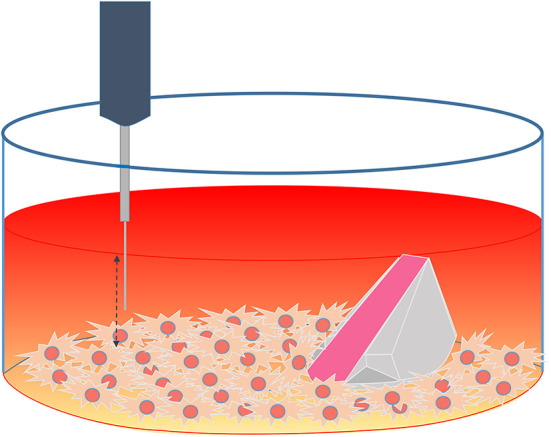
Schematic representation of the experimental set up using both techniques (ramp and microsensor) inside a confluent cell culture well.

An optical fiber-based needle type sensor PM-PSt7 (PreSens, Regensburg, Germany) mounted on a motorized Automated Micromanipulator (AM, PreSens, Regensburg, Germany) was employed for measuring the oxygen concentration at different heights inside one of the confluent cell culture well after 96 h of measurements with the ramps and the VisiSens TD system (Figure 3).

Setting up the PM-PSt7 implies that the culture plate remains open during the measurements. To prevent contamination and media evaporation, the wells were covered with a plate seal foil Microseal “B” adhesive seal (Bio-Rad Laboratories, Germany) and perforated with the needle type sensor. The diameter of the optical fiber of the PM-PSt-7 probe is <50 um and the diameter of the needle is 0.8 mm.

The micromanipulator was set to move the PM-PSt7 sensor through the cell culture medium along the z-axis with a step size of 500 μm. Sets of six measurements with an interval of 10 s between measurements were performed at six different altitudes. At each altitude, the micromanipulator stopped its movement and, after a resting time of 30 s, the set of measurement for that altitude was performed. The resting time of 30 s prior starting the measurement at each altitude was included to avoid measurements in the presence of turbulences created by the movement of the microsensor through the cell culture medium. Data were collected by means of the software PreSens Profiling Studio (PreSens, Regensburg, Germany).

The sterilization of the PM-Pst7 micro sensor was conducted by incubation in 70% ethanol for 30 min fallowed by washing steps with PBS.

### Statistical Analysis

The statistical analysis was performed by means of the GraphPad Prism 8.3.0 software (GraphPad Software, Inc., La Jolla, USA). For comparison of the oxygen profiles, a minimum of *N* = 3 measurements were considered. Two-way ANOVA associated with Tukey's multiple comparison test was selected for evaluation of the oxygen profiles at different time points per cell density. Linear regression was performed for the oxygen gradients obtained after 96 h of culture for the three different cell densities.

## Results

### Non-invasive Oxygen Concentration Measurement at Cell Monolayer

**Figure 4:**
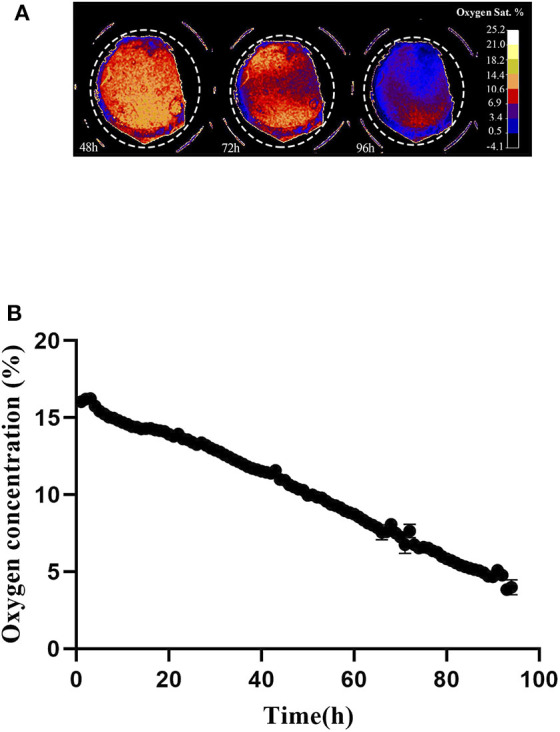
**(A)** Output from the VisiSens ScientificCal software after calibration and noise reduction. 7 × 10^4^ cells where seeded in a 48 well plate containing the SF-RPsSu4 sensors attached to the bottom of the wells. Images were taken every hour for a period of 96 h. Time points 48, 72, and 96 h are shown. Dashed circle indicate the selection of the ROI. **(B)** Z-Profile of the oxygen concentration measured at the cell layer by means of the VisiSens TD system and the SF-RPsSu4 sensor foils attached to the bottom of the well. Initial cell density 7 × 10^4^ cells/cm^2^.

Oxygen depletion at the cell layer was monitored by means of the VisiSens system during 96 h, starting immediately after cell seeding. Figure 4A shows a preview from the VisiSens's output once noise reduction and calibration were applied for three different time points of interest; 48, 72, and 96 h. As shown in Figure 4B, oxygen concentration at the cell layer steadily decreased from 16 to 3% during the time in culture. The obtained data confirm that during cultivation, oxygen concentration at the cell layer decreases as a result of normal respiration.

### Non-invasive Oxygen 3D Gradient Measurement

**Figure 5:**
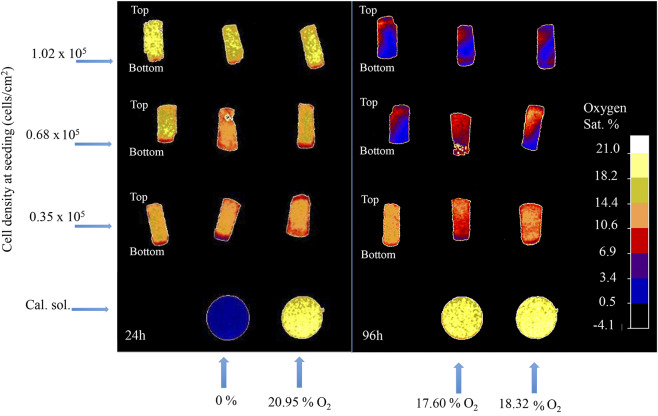
Software output for two different time-points after seeding; 24 and 96 h. Rounded shaped signals corresponds to the calibration solutions. Rectangular shaped signals correspond to the sensors attached to the surface of the ramps.

**Figure 6:**
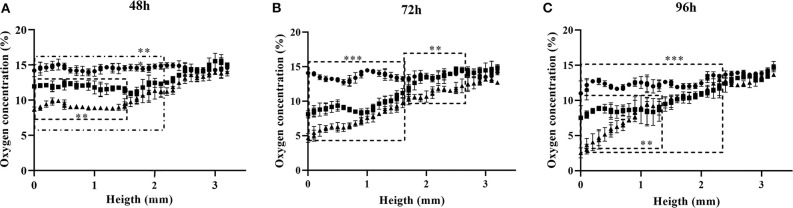
Oxygen profiles obtained for three different cell densities at time points 48 **(A)**, 72 **(B)**, and 96 h **(C)** after seeding. Seeding densities are: • 3.5 × 10^4^ cells/cm^2^; ■ 6.8 × 10^4^ cells/cm^2^; ▴ 1.02 × 10^5^ cells/cm^2^. Significant differences (*p* < 0.0001) between profiles are delimited.

The 3D oxygen gradient in the cell culture medium column over the cell layer was effectively measured in a non-invasive manner by means of the ramps and the VisiSens TD camera system. Figure 5 shows a preview of the output from the VisiSens TD system for two different time points, 24 and 96 h after seeding. The obtained oxygen profiles (the oxygen concentration as function of the height of the cell culture medium column above the cell layer) for each initial cell density and three time points after seeding are shown on Figure 6. The acquired profiles show clear differences in the oxygen distribution within the cell culture medium column between each cell density per time point. Significant differences (*p* < 0.0001) were observed between the oxygenation values measured at different heights in the cell culture medium column for each cell density at the three selected time points; 48, 72, and 96 h. An overview of the differences in the oxygen distribution at the top and the bottom of the ramps as well as the obtained OCR for the cells after 96 h in culture are summarized in Table 1. The OCR values obtained after 96 h of culture were 96.3 ± 12.53 and 107 ± 10.17 fmol/h/cell, respectively for the wells with higher cell density, where a theoretically linear oxygen gradient (*R*^2^ > 0.9) was observed. The oxygen gradient measured for the wells with the lowest initial cell count after 96 h of culture rendered values of *R*^2^ <0.7 and therefore, accurate calculation of OCR was not possible for these wells.

**Table 1:** Oxygen concentration (%) during time in culture.

**Initial cell density (× 10^**5**^ cells/cm^**2**^)**		**48 h**	**72 h**	**96 h**	**Cell density at 96 h (× 10^**5**^ cells/cm^**2**^)**	***R*^**2**^ (top to bottom linearity)**	**OCR at 96 h of culture (fmol/h/cell)**
0.35	Top	14.58 ± 0.49	14.18 ± 0.37	14.8 ± 0.74	1.57 ± 0.32	0.66	–
	Bottom	14.02 ± 0.88	13.28 ± 0.28	11.01 ± 1.21			
	Difference	0.56	0.9	3.79			
0.68	Top	15.04 ± 0.23	14.89 ± 0.42	14.69 ± 0.39	2.8 ± 0.49	0.91	96.3 ± 12.53
	Bottom	11.95 ± 0.22	8.10 ± 0.52	7.52 ± 0.12			
	Difference	3.09	6.79	7.17			
1.02	Top	14 ± 0.57	12.91 ± 0.25	13.66 ± 0.1	3.42 ± 0.67	0.93	107 ± 10.17
	Bottom	8.71 ± 0.32	4.67 ± 0.63	2.53 ± 0.71			
	Difference	5.29	8.24	11.13			

*Comparison of the pericellular oxygen concentration (base of the ramp) and the concentration at the top of the ramp (3.3 mm from the base) for A549 cells cultured at 18.5% oxygen in 24 well plates. Three initial cell densities were seeded (n = 3). One plate was used for the oxygen measurements and one was used to determine the cell count at each time point. After 96 h of culture, linear regression was performed and oxygen consumption rates (OCR) was calculated from the obtained oxygen gradients with R^2^ > 0.9*.

After 48 h of culture, significant differences (*p* < 0.0001) were observed for the oxygen values measured, from the bottom to an approximated height of 2.2 mm over the cell layer between the wells seeded with 5 × 10^4^ cells and those with higher cell densities. Meanwhile, only significant differences (*p* < 0.0001) were found for the measurements from the bottom to a height of 1.5 mm between the wells seeded with 1 × 10^5^ and 1.5 × 10^5^ cells (Figure 6A). For the next time point of 72 h, significant differences (*p* < 0.0001) were measured for the oxygenation values from the bottom to an approximate height of 1.7 mm between the three different cell densities. From this height and to the top of the ramp, only significant differences were found between the wells seeded with the higher cell count of 1.5 × 10^5^ cells and the other two cell densities (Figure 6B). Ninety-six hours after seeding, significant differences (*p* < 0.0001) for the oxygen values measured at each point below a height of 2.5 mm from the bottom of the well were found between the wells seeded with the lower cell count and the other two (higher) cell densities. At this point, the wells seeded with 1 × 10^5^ and 1.5 × 10^5^ cells, reported significant differences (*p* < 0.0001) from the bottom to a height of 1.2 mm over the cell layer. Above this height to the top of the ramp, the profiles obtained for both cell densities were indistinct (Figure 6C).

### Oxygen Profiling With Fiber-Based Needle Type Microsensor

The PM-PSt7 sensor was employed to measure the oxygen profile after 96 h of measurement with the ramps and the VisiSens TD system. Oxygenation values at the top and the bottom of the well-obtained with the two techniques are summarized in the Table 2. No significant differences were found between the obtained profiles from the two techniques.

**Table 2:** Comparison of the oxygen gradient (%) obtained by two different techniques.

	**VisiSens + 3D printed ramp**	**Microsensor PSt7**
Top	12.9 ± 0.39	10.15 ± 0.49
Bottom	7.52 ± 0.17	6.2 ± 0.9
Difference	5.3	4.2

*After 96 h of measurement with the VisiSens TD camera system, the micro sensor PM-PSt7 was employed to measure the oxygen gradient in one of the confluent wells previously studied by means of our ramp-based technique. n = 3*.

## Discussion

A complex system of organs and tissues work together *in vivo* to finely tune oxygen concentration and distribution across temporal and spatial scales (Wittenberg and Wittenberg, 1989; Saltzman et al., 2003; Al-Ani et al., 2018). On the other hand, diffusive forces mainly driven by the oxygen consumption rate of the cells and the diffusion properties of cell culture media are the major regulatory mechanism for oxygenation during cell culture.

The limitation of oxygenation in cell culture stands as one of the most underappreciated problems of basic and translational research (Place et al., 2017; Ast and Mootha, 2019) potentially having a direct impact not only on the experimental results but in their reproducibility (Begley and Ellis, 2012; Begley and Ioannidis John, 2015; Karp, 2018). This is especially relevant in the fields of regenerative medicine and tissue engineering where so much effort is focused on the design of materials and scaffolds to provide with the adequate oxygen supply to the newly formed tissue (Fiedler et al., 2014).

The “conventional” technique for oxygen sensing involving the VisiSens TD camera system and the SF-RPsSu4 sensor foils at the bottom of the wells proved during our studies, to be a suitable tool for non-invasive measurements of the variation in the oxygen concentration close to the surface of the cells. Nonetheless, no relevant information about 3D oxygen gradient formation in the cell culture medium column above the cell layer could be obtained with the conventional set up.

Similar limitations would be observed using other examples of the current commercially available technology for oxygen sensing. For instances, the RED eye patch oxygen sensor works in combination with the Neofox fluorometer and a fiber optic probe to measure the concentration of oxygen in gas, as well as dissolved oxygen in solutions. The output of this system provides a single oxygen concentration values per measurement. This feature makes this system suitable for pharmaceutical and medical application, such as blood bag analysis or bioprocess control. Nevertheless, it make it inadequate when it comes to measuring oxygen gradients in cell culture. Furthermore, multiplexing of RED eye patches for simultaneous measurements would be limited to low numbers of sensors due to the involvement of fiber optic probes per sensor foil necessary for the measurements (Molina et al., 2014; Gandolfo et al., 2016).

With our developed method, it was possible to assess how the oxygen tension at the cell layer decreases as a function of cell density and time in culture in a non-invasive fashion. The absence of moving parts in our set up ensures that the oxygen distribution inside the cell culture well is due only to the cellular respiration and the oxygen diffusion through the cell culture medium. Medium exchange was not conducted to avoid external disturbances to the oxygen distribution in the stagnant cell culture medium column. The cell line A549 was selected for this study because of its wide range of application as a model in cancer research and lung diseases (Schnitzer et al., 2009; Jeong et al., 2017; Camerlingo et al., 2019). Moreover, according to previous studies, the expansion and proliferation of this cell line does not appear to be significantly inhibited by cell contact when cultured in DMEM medium (Cooper et al., 2016). Thus, high cell density culture can be easily achieved in relatively short periods of time (Cooper et al., 2016).

Attaching the SF-RPSu4 sensor foil on the 3D printed ramp effectively allowed the interaction between the sensor and the oxygen molecules at different height at the cell culture medium column. The utilization of the VisiSens TD camera system permitted the non-invasive real-time oxygen monitoring from the moment of seeding to the end of the experiment 96 h later. Few differences in the oxygen distribution inside the wells with lower cell count after 96 h of culture were observed. For these wells, oxygen was found to range from 11 to 15% from the bottom to the top of the ramp at the latest time point. Nevertheless, it is worth noticing that the oxygen tension measured at the bottom of the wells after 96 h of culture (11%) is considerably lower than the oxygen concentration in the gas phase of the incubator (18.5%).

For the wells seeded with 1 × 10^5^ and 1.5 × 10^5^ cells, respectively, significant differences (*p* < 0.0001) between the oxygen distributions within the cell culture medium column were also observed. These differences were especially noticeable during the first 72 h of culture when the biggest differences in cell density between the wells with different initial cell density are to be observed. However, by the end of the measurements, oxygen profiles for both cell densities were found to be very similar. Only significant differences were registered within a distance of 1 mm from the bottom of the well. This is expected to be related to the cell count at each time point. For instance, at 72 h of culture the wells seeded with 1.5 × 10^5^ cells (superconfluent by time point 96 h) achieved 35% higher cell density than those seeded with 1 × 10^5^ cells (confluent by time point 96 h). Meanwhile, after 96 h of culture, the difference between cell densities was only of 18%. This is to be expected as it is known that highly dense cultures tend to reduce expansion rate when nutrients in the culture media are depleted (Vander Heiden et al., 2009; Yuan et al., 2013)

The OCR for the A549 cell line calculated using the obtained oxygen gradients and cell count after 96 h of culture from the highly confluent and superconfluent wells show no significant differences and are very similar to the OCR values reported in the literature for this cell line, which is 97 fmol/h/cell (Wu et al., 2007; Wagner et al., 2011). OCR determined from the gradient measured in the subconfluent wells was not considered due to the lower *R*^2^ values. This could be related to the fact that, during cultivation of low cell densities cultures, the oxygen consumption by the cell monolayer tends to occur just fast enough to barely surpass the oxygen ability to diffuse into the cell culture medium and to the bottom of the well (Place et al., 2017; Al-Ani et al., 2018). Based on this, we hypothesize that, even when the oxygen concentration at the bottom of the well was registered to be lower than the oxygen concentration of the gas phase, the oxygen consumption rate of the cell monolayer in low cell density culture was not sufficient to promote a clear tendency to the formation of a linear gradient of oxygen concentration, hence, rendering lower values of R^2^.

The statistical analysis of the comparison between the oxygen gradient measured with the 3D printed ramps and the PM-PSt7 sensor after 96 h of culture showed no significant differences. Interestingly, oxygen concentration closer to the surface of the cell culture medium was measured to be 12.9% when employing the VisiSens technique with the 3D printed ramps. Meanwhile, a value of 10.15% was obtained with the PM-Pst7 microsensor (Table 2). Such a difference of 3 percentage units between the obtained oxygen concentration values from each technique was reduced to only 1 percentage unit at the bottom of the well and closer to the cell layer. The lower oxygen concentration at the surface of the cell culture medium measured with the PM-Pst7 microsensor could be related to the utilization of the plate seal foil Microseal “B” to cover the cell culture well during the oxygen measurement with the microsensor. This foil was used to prevent evaporation of the cell culture medium and minimize the risk of contamination during the measurements. Yet, the low permeability to oxygen declared by the manufacturer for the Microseal “B” could have created a “closed environment” during the measurements with the microsensor that modified the oxygen concentration in the gas phase and the surface of the cell culture medium. This possible change in the atmosphere of the cell culture well was not expected to affect the oxygen concentration closer to the cells in culture during the oxygen measurement with the microsensor due to the fact that the required time for performing the necessary measurements, does not exceed 30 min and the equilibration of oxygen in culture medium is known to be a slow process that requires significantly longer time periods (Allen et al., 2001; Place et al., 2017).

The obtained result validates the use of our 3D printed ramps as a suitable tool for measuring oxygen concentration at different heights at the cell culture medium column. Moreover, in contrast to the microsensor measurements, our ramps allowed the study of several culture wells simultaneously while keeping sterile conditions during the measurement. Furthermore, our non-invasive, optical sensor foil based technique for oxygen measurement during cell culture, does not involve any moving parts or cables and allowed the study of the complete gradient formation, from the bottom of the cell culture well to the surface of the well with micrometric resolution. The use of the SF-RPSu4 sensor foils has been validated since more than 10 years of research and reported in over a hundred scientific publications (Kellner et al., 2002; Faget et al., 2013; Hofmann et al., 2013; Maisch et al., 2019; Wolff et al., 2019). The self-adhesive sensor foils can be attached to the ramps very easily. The dye molecules inside the sensitive layer of the SF-RPSu4 sensor foil are incorporated in a polymer matrix and have no contact to the washing solutions, therefore cleaning of both, ramps and sensor foils can be conducted with ethanol 70% prior the beginning of the measurements. Furthermore, the sensor's response is fully reversible and shows no signal hysteresis, which means that these sensors can be used repeatedly. Nevertheless, a check of the calibration data is performed after ~10,000 images.

Additionally, the sensor foils used on the ramps comprise a hydrophobic polymer surface material that hampers cell attachment without prior treatment. This way the growth of undesired biofilms on the sensor that could lead to biofouling is prevented. Besides, the ramp puts the sensor in an angle to the cell culture bottom of the well plate, which makes it more likely for biological material to sediment to the bottom and attach there and not to the foil on the ramp. Not to mention that adherent cells of a size of 10–15 μm (A549 cells) cannot grow up the ramps which has a size of several millimeters.

This makes our system a suitable tool as first solution to easily check a protocol over the whole time period of incubation with respect to sufficient/adequate oxygen supply. Moreover, due to the simplicity of our setup, oxygen gradient measurements are possible in any standard cell culture plate (96, 48, 24, 12, 6 well plate) allowing the study of oxygenation during cell culture in different conditions (e.g., difference in medium volume, height of column medium and cell concentration) while monitoring a number or even all wells of a cell culture plate simultaneously. This feature allows, not only to ensure that oxygenation conditions are replicated while scaling cell culture/experiments from one plate to another of different size, but it also improves the reproducibility of the experiments by providing a tool to easily report the oxygenation condition at the moments of the performed assays.

During our study, we have used the ramps with the cell line A549 as proof of concept. Nevertheless, we expect our system to be reliable when applied to a different cell line. Of course, cell lines with lower growth rate and/or metabolic activity are expected to induce less steep gradients during culture in the same period than cell lines with higher growth rates (assuming same initial seeding density). Similarly, cell lines with low metabolic activity may need higher cell densities or longer time in culture to deplete the oxygen near the surface of the cells faster than the diffusion rate of the oxygen to the bottom of the well. Answering this kind of questions is one of the applications for the developed technique.

Furthermore, future applications for our technique could involve the adaptation of other types of sensor foils (e.g., pH and CO_2_) that are compatible with the VisiSens TD camera system (Blossfeld et al., 2013; Keil et al., 2017) to the developed ramps together with the SF-RPSu4 sensor foil. The development of a multimodal sensor tool to study in a non-invasive manner the gradient and distribution of not only oxygen, but CO_2_ and pH, could provide a higher throughput analysis of cell respiration and metabolic activity. Tracking of the CO_2_ released per unit of O_2_ consumed could allow the determination of the respiratory quotient while the monitoring of the pH allows the accurate quantification of the dissolved CO_2_ (Keil et al., 2017), Nonetheless, an inconvenience of utilizing the ramps for oxygen sensing during cell culture is the 54 mm^2^ surface area occupied by the ramp inside the culture well. This means that, from the 200 mm^2^ available for cell growth in a 24 well plates, only 146 mm^2^ are open for the cells. This needs to be considered when calculating the cell density per cm^2^. Optimization in terms of design of the ramp's geometry should be conducted to reduce the total area in contact with surface of the wells.

## Conclusions

With the present study we aimed to the development of a simple, cost-effective technique for non-invasive 3D oxygen gradient measurement during mammalian cell culture. For this, we created a 3D printed ramp to use in conjunction with the optical sensor foil SF-RPSu4 and the VisiSens TD camera system. With our developed technique, it was possible to measure the oxygen gradient formation during cell culture of A549 cells at different cell densities simultaneously in a non-invasive way. The data gathered from the oxygen distribution inside the cell culture medium column was used to calculate the oxygen consumption rate of the cells in culture and the obtained value matches those reported in the literature. Thus, our optical sensor foil-based technique could be utilized for characterization of oxygen consumption rates of different cells types. Furthermore, the 3D oxygen gradient measured with the ramps was comparable to that measured with the needle type microsensor. Additionally, our technique was found to be unique in its ability to monitor simultaneously the 3D oxygen gradient formation in several wells of a culture plate. Future applications for our 3D printed ramps could as well involve testing other different sensor foils, such as pH or CO_2_ optical sensor foils.

## Data Availability Statement

The raw data supporting the conclusions of this article will be made available by the authors, without undue reservation.

## Author Contributions

CP performed all the experiments, acquired the data, performed the data analysis, and wrote the first version of the manuscript. GL, RM, and MSG designed and produced the ramps, participated in the study design, and supported all the experiments performed. EB participated in the experimental design, data analysis, and statistics. MvG participated in the experimental design and statistical analysis, directed the study, and critically revised the manuscript. All authors contributed to the article and approved the submitted version.

## Conflict of Interest

GL, RM, and MSG are employees of PreSens Precision Sensing GmbH, Regensburg, Germany. The remaining authors declare that the research was conducted in the absence of any commercial or financial relationships that could be construed as a potential conflict of interest.
